# 

**Published:** 1895-10

**Authors:** 


### TABLE OF CONTENTS.

ORIGINAL COMMUNICATIONS. PAGE

Pyorrhœa Alveolaris. By Dr. J. E. Cravens, Indianapolis, Ind. . . . . 591

Further Experience with Balsamo del Deserto. By Dr. W. H. White, Silver City, N. M. . . . . . . . . . . . . . . . . . . . . . . . . 601

The Thorough Removal of Deposits in the Successful Treatment of Pyorrhœa. By Dr. James H. Daly, Boston . . . . . . . . . . . . . . . 605

The Dental Student. By William W. Belcher, D.D.S., Seneca Falls, N. Y. . . . . . . . . . . . . . . . . . . . . . . . . . . . 608

REPORTS OF SOCIETY MEETINGS.

American Dental Association . . . . . . . . . . . . . . . . . . . 611

American Academy of Dental Science . . . . . . . . . . . . . . . . 630

National Association of Dental Examiners . . . . . . . . . . . . . . 638

EDITORIAL.

The Recent Conventions . . . . . . . . . . . . . . . . . . . . . 641

The "Faculties" and Honorary Degrees . . . . . . . . . . . . . . . 646

Erratum . . . . . . . . . . . . . . . . . . . . . . . . . . . 647

BIBLIOGRAPHY . . . . . . . . . . . . . . . . . . . . . . . . 647

OBITUARY.

Dr. Edwin C. Baxter . . . . . . . . . . . . . . . . . . . . . . 648

Sir John Tomes . . . . . . . . . . . . . . . . . . . . . . . . 649

NOTES AND COMMENTS . . . . . . . . . . . . . . . . . . . . 651

CURRENT NEWS.

Union Dental Meeting . . . . . . . . . . . . . . . . . . . . . . 653

Dental Association of New South Wales . . . . . . . . . . . . . . . 653

Southern Dental Association . . . . . . . . . . . . . . . . . . . 654

American Dental Society of Europe . . . . . . . . . . . . . . . . . 654

